# A Comparative Study on the Fracture Resistance of CAD/CAM–Fabricated Single‐Piece Post‐Crowns

**DOI:** 10.1002/cre2.70247

**Published:** 2025-12-15

**Authors:** Ali Erdem, Mehmet Selim Bilgin, Ibrahim Ersoy, Erhan Dilber, Ebru Nur Işık, Tan Fırat Eyüboğlu, Mutlu Özcan

**Affiliations:** ^1^ Private Practice İzmir Türkiye; ^2^ Faculty of Dentistry, Department of Prosthodontics Alanya Alaaddin Keykubat University Alanya Türkiye; ^3^ Private Practice İstanbul Türkiye; ^4^ Department of Endodontics, Faculty of Dentistry Istanbul Medipol University Istanbul Türkiye; ^5^ Clinic of Masticatory Disorders and Dental Biomaterials, Center for Dental Medicine University of Zurich Zurich Switzerland

**Keywords:** CAD/CAM, dental materials, fracture resistance, polymer‐infiltrated ceramic network, post‐crown, Yttrium tetragonal zirconia polycrystal

## Abstract

**Objectives:**

Recently, CAD/CAM materials have become popular in dental clinics; however, information about their fracture resistance as post‐crowns remains limited. This study compared the initial fracture resistance of potential single‐piece post‐crown materials made with CAD/CAM milling to sound teeth.

**Materials and Methods:**

Fifty freshly extracted, non‐carious human central incisor teeth underwent endodontic treatment. The roots were then randomly divided into five groups based on the post systems: the control group included teeth filled only with gutta‐percha. Monoblock post‐crowns were made using four different systems for the other groups: LDS (IPS e.max CAD®, lithium disilicate glass‐ceramic), YTZP (inCoris ZI®, Yttrium tetragonal zirconia polycrystal), RNC (Lava Ultimate®, resin‐based nanoceramic), and PICN (VITA Enamic®, polymer‐infiltrated ceramic network). The post‐crowns were cemented with resin cement and tested with a universal testing machine at a crosshead speed of 1.0 mm/min. Data analysis used one‐way ANOVA and multiple comparison post‐hoc Tukey tests (*α* = 0.05).

**Results:**

Significant differences were found between the groups (*p* < 0.001). The control group exhibited the highest fracture resistance (749.25 ± 225.02 N). YTZP showed similar resistance to the control (*p* = 0.99) and LDS (447.28 ± 168.72 N, *p* = 0.081), but was significantly higher than RNC (343.79 ± 157.08 N, *p* = 0.0051) and PICN (348.78 ± 157.44 N, *p* = 0.0059). LDS, RNC, and PICN did not differ significantly. YTZP experienced more non‐repairable fractures (5/10), while PICN predominantly failed in a repairable manner (9/10).

**Conclusions:**

All CAD/CAM post‐crowns exceeded functional loads for the anterior region. YTZP and LDS demonstrated greater strength, whereas PICN and RNC, despite being weaker, favored repairable failures—highlighting the importance of balancing strength and clinical manageability.

## 1 Introduction

The final restoration of endodontically treated teeth (ETT) should achieve adequate aesthetics, function, and protection (Mortazavi et al. 2012). Strength and aesthetics influence the choice of treatment for ETT (Bittner et al. 2010). When the coronal structure of ETT is compromised, post and core systems are preferred to support the final restoration. The primary role of post systems is to provide retention for the core and coronal structure while replacing the damaged coronal tissues (Vafaee et al. 2010). Various materials have been utilized to fabricate posts, including custom and prefabricated metals and non‐metals (Bateli et al. 2014). Custom‐cast metal post‐cores have been effectively used for decades to restore ETT (Salvi et al. 2007). However, metal posts may cause corrosion reactions, leading to a metallic taste, oral burning, pain, and even allergic reactions (Shetty et al. 2013). More biocompatible and aesthetic restorations emerged in the late 1980s as prefabricated glass‐ceramic posts and core buildups (Bateli et al. 2014; Christel et al. 1989). In 1995, Pissis (1995) introduced a “monoblock” technique for a post, core, and crown made from glass‐ceramic, and that same year, zirconia ceramic was introduced to address the low fracture strength of glass‐ceramic material (Meyenberg et al. 1995).

Over the past decade, advancements in CAD/CAM technology have enabled the production of custom zirconium dioxide and glass‐fiber posts and cores (Awad and Marghalani 2007; Lee 2014). The partially sintered zirconium blocks are intended for crafting crown copings and anterior and posterior fixed partial denture frameworks with up to two pontics. Initially, the frameworks are milled to a slightly larger size. After the sintering process, they obtain the required properties. High‐strength lithium disilicate, translucent zirconium dioxide ceramics, and aesthetic CAD/CAM block ceramics are produced through CAD and machining with two stages of buildup to achieve their final high strength (Mörmann et al. 2013). Resin nanoceramic (RNC) blocks represent a significant breakthrough in CAD/CAM dentistry, as they enhance aesthetics by permitting the use of composite materials for fine adjustments after milling. Combining composite materials to a final restoration is feasible with nano‐ceramic particles embedded in a highly cured resin matrix (Koller et al. 2012). With the enduring aesthetics of ceramics, the newly introduced polymer‐infiltrated ceramic network (PICN) material offers an alternative solution, and due to its comparable Young's modulus to dentin, it serves as an ideal restorative material for post and crown techniques (Della Bona et al. 2014).

PICN structures are thought to closely mimic the properties of natural teeth more than current restorative materials (Coldea et al. 2013). However, the fabrication of post‐crowns using these new restorative materials has not been extensively studied. This study aimed to assess whether these materials possess sufficient durability for use as post‐crowns. The null hypothesis (H0) stated that there is no significant difference in the fracture resistance between teeth with intact coronal structure and teeth restored with post‐crowns made from various CAD/CAM materials.

## 2 Materials and Methods

Considering four different CAD/CAM post‐crown groups and one method for each, power analysis using G*Power (Faul et al. 2009) indicated an actual power value of 80, with 10 specimens per group, based on the following parameters: effect size (*f* = 1), *α* = 0.005; power = 80; noncentrality parameter = 15; and critical *t* = 5.

A total of 50 non‐carious human central incisor teeth, freshly extracted due to periodontal disease, were stored in 0.9% isotonic saline solution (Eczacıbaşı, Istanbul, Türkiye) before use. The calculus and remnants were removed from the surfaces of the teeth. Aside from the control group, the roots of the teeth were removed at the cemento–enamel junction, 13 mm from the root apex, using a diamond disc (KG Sorensen, Barueri, SP, Brazil). After separating the crowns from the roots, the root canals were prepared to the level of a #40 file using Reciproc rotary instruments (VDW GmbH, Munich, Germany) with 1 mL of 2.5% sodium hypochlorite (NaOCl) (Wizard, İzmir, Türkiye) used for irrigation between each file. For the final irrigation protocol, 3 mL of 2.5% NaOCl was applied for 30 s, followed by 17% ethylenediaminetetraacetic acid (EDTA) solution (Wizard, İzmir, Türkiye) for 1 min, and distilled water (Aqua Dest, B. Braun, Melsungen, Germany) for 30 s. The canals were dried with absorbent paper points (Dentsply Maillefer, Ballaigues, Switzerland).

All root canals were obturated using the cold lateral compaction technique with an epoxy resin‐based sealer (AH Plus; Dentsply DeTrey, Konstanz, Germany) and gutta‐percha cones (Dentsply Maillefer, Ballaigues, Switzerland), then sealed with temporary filling material (Cavit, 3M ESPE, Seefeld, Germany).

In the control group, the cavities of the teeth were filled with nanohybrid composite resin (G‐aenial, GC Corp., Tokyo, Japan), and no other preparation was performed. All samples were stored in distilled water (Aqua Dest, B. Braun, Melsungen, Germany) at 37°C for 7 days, after which 8 mm of the root canal filling was removed with a Peeso reamer bur (Dentsply Maillefer, Ballaigues, Switzerland) operating at low speed, leaving 4 mm of gutta‐percha within the root canal. The root canals were washed with 3 mL of 2.5% NaOCl, rinsed with 10 mL of distilled water, and dried with sterile paper points.

Impressions for each specimen's root canal were taken using polyvinyl siloxane impression material (Elite HD+, Zhermack SPA, Badia Polesine, Italy) and sent to a technician to be scanned with a high‐definition laboratory scanner (inEos X5, Sirona Dental Systems GmbH, Bensheim, Germany). The 3D image datasets of the specimens were transferred to the InLab CAD software (InLab SW 4.2, Sirona Dental Systems GmbH, Bensheim, Germany) for designing and building post‐crowns with dedicated blocks.

**Figure 1:**
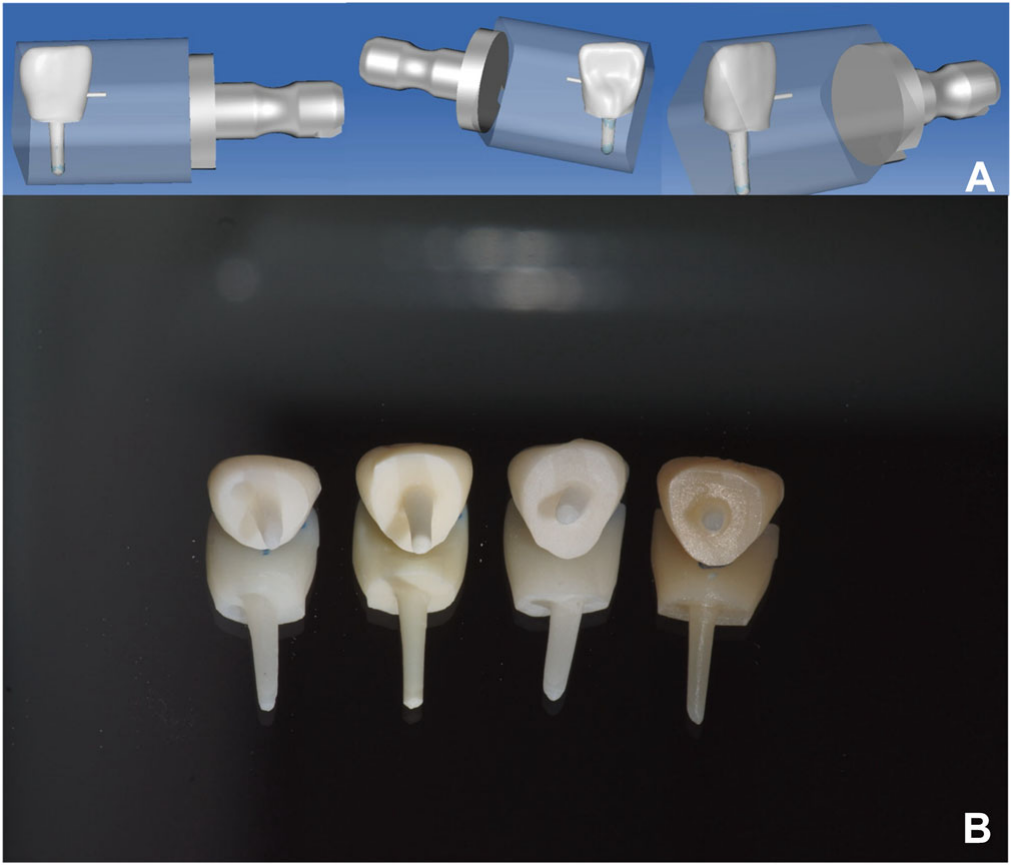
A. Software design of CAD/CAM post crowns. B. A representative image of prepared CAD/CAM post crowns. From left to right: LDS (IPS e.max CAD), YTZP (inCoris ZI), RNC (Lava Ultimate), and PICN (VITA Enamic).

The post‐crown specimens were divided into four groups (*n* = 10 per group) for designing and fabricating with four different materials: LDS (IPS e.max CAD, Ivoclar Vivadent AG, Schaan, Liechtenstein); YTZP (inCoris ZI, Sirona Dental Systems GmbH, Bensheim, Germany); RNC (Lava Ultimate, 3M ESPE, St. Paul, MN, USA); and PICN (VITA Enamic, VITA Zahnfabrik H. Rauter GmbH & Co. Bad Säckingen, Germany) (Table 1, Figure 1A,B).

**Table 1:** Composition and manufacturer details of CAD/CAM materials and adhesives used in this study.

Material	Brand/Manufacturer	Chemical composition
Lithium disilicate glass ceramic	IPS e.max CAD/Ivoclar Vivadent, Schaan, Liechtenstein	57%–80% SiO_2_, 11%–19% Li_2_O, 0%–13% K_2_O, 0%–8% other oxides
Polymer‐infiltrated ceramic network (hybrid ceramic)	Vita ENAMIC/Vita Zahnfabrik, Bad Säckingen, Germany	58%–63% SiO_2_, 20%–23% Al_2_O_3_, 6%–11% Na_2_O, 4%–6% K_2_O, 0.5%–2% B_2_O_3_, < 1% CaO, < 1% TiO_2_
Yttrium tetragonal zirconia polycrystal	inCoris ZI/Sirona Dental Systems GmbH, Bensheim, Germany	≥ 99.0% ZrO_2_ + HfO_2_ + Y_2_O_3_, > 4.5–≤ 6.0% Y_2_O_3_, ≤ 5% HfO_2_, ≤ 0.5% Al_2_O_3_, ≤ 0.3% Fe_2_O_3_
Resin nanoceramic	Lava Ultimate CAD/CAM/3M ESPE, St. Paul, MN, USA	Bis‐GMA, UDMA, BIS‐EMA, TEDGMA. Filler: SiO_2_, ZrO_2_, Si/ZrO_2_ cluster, 80% by weight
Universal adhesive	Single Bond Universal/3M ESPE, St. Paul, MN, USA	2‐HEMA, 10‐MDP, dimethacrylate resins, Vitrebond, copolymer, silane, filler, ethanol, water, initiators, pH: 2.7

The resulting post‐crowns were cleaned with 70% ethanol (Merck KGaA, Darmstadt, Germany), rinsed with distilled water (Aqua Dest, B. Braun, Melsungen, Germany), and then air‐dried. All groups were cemented with self‐adhesive resin cement (RelyX Ultimate Clicker, 3M ESPE, St. Paul, MN, USA), and an adhesive containing 10‐MDP monomer (Single Bond Universal + DCA activator, 3M ESPE, St. Paul, MN, USA) was applied according to the manufacturer's recommendations (Table 1).

A specimen in group PICN was etched for 60 s with 9.6% hydrofluoric acid gel (Ultradent Porcelain Etch, Ultradent Products Inc., South Jordan, UT, USA), while group LDS was etched for 20 s with 5% hydrofluoric acid gel (IPS Ceramic Etching Gel, Ivoclar Vivadent AG, Schaan, Liechtenstein). Samples in groups YTZP and RNC were sandblasted with 30‐µm silica‐coated alumina particles (CoJet Sand, 3M ESPE, Seefeld, Germany) at 2 bar pressure. Following these steps, all post‐crown groups were rinsed with distilled water for 20 s and then dried with oil‐free compressed air (Dürr Dental, Bietigheim‐Bissingen, Germany).

**Figure 2:**
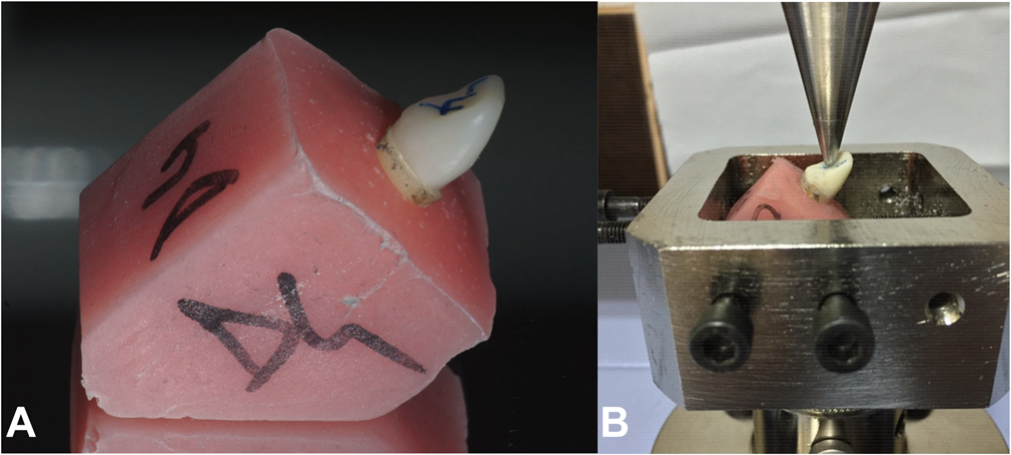
A. A representative image of a prepared sample. B. The placement of the sample in the universal testing machine.

An area was marked for loading on the lingual surfaces of the teeth or crowns. The samples were tested with a universal testing machine (Autograph AGS‐X, Shimadzu Corporation, Kyoto, Japan) using a 5 kN load cell (Shimadzu Corporation, Kyoto, Japan) at a crosshead speed of 1.0 mm/min to record initial fracture resistance. All samples were embedded in self‐curing acrylic resin blocks (Orthocryl, Dentaurum, Ispringen, Germany) arranged to fit into the loading location of the universal testing machine at a 45° angle to the horizontal plane (Figure 2A,B).

In addition, all fractures were examined under visual inspection and classified into two main categories: repairable (post or coronal fractures above the cemento–enamel junction) and non‐repairable (vertical root fractures or catastrophic fractures extending below the cemento–enamel junction) as described previously (Ozcopur et al. 2010; Ramesh et al. 2016; Vartak et al. 2025).

The normality of the data was assessed using the Shapiro–Wilk test (SPSS Statistics v25.0, IBM Corp., Armonk, NY, USA). One‐way ANOVA and post‐hoc Tukey tests were employed to analyze the statistical data.

## 3 Results

The control group exhibited the highest mean fracture resistance, measuring 749.25 ± 225.02 N. One‐way ANOVA revealed significant differences among the groups (*p* < 0.0001). The post‐hoc Tukey test indicated no significant differences between the control and YTZP groups (*p* = 0.99) or between LDS and YTZP groups (*p* = 0.081). Furthermore, the control group showed significantly higher fracture resistance compared to the LDS (447.28 ± 168.72, *p* = 0.027), RNC (343.79 ± 157.08, *p* = 0.0013), and PICN (348.78 ± 157.44, *p* = 0.0015) groups, respectively. The YTZP group also demonstrated significantly higher fracture resistance than the RNC (*p* = 0.0051) and PICN (*p* = 0.0059) groups, but not compared to LDS (*p* = 0.081). The LDS, RNC, and PICN groups did not present statistically significant differences from one another (*p* > 0.05) (Table 2). All specimens exhibited no signs of pre‐failure during handling or before testing, with fractures occurring solely under controlled loading conditions.

**Table 2:** Fracture resistance values (mean ± standard deviation) for each group.

Group	*n*	Mean ± SD (N)
Control	10	749.25 ± 225.02^a^
LDS	10	447.28 ± 168.72^ab^
YTZP	10	668.87 ± 336.60^a^
RNC	10	343.79 ± 157.08^b^
PICN	10	348.78 ± 157.44^b^

*Note:* Groups with the same superscripted letters indicate no statistically significant difference at *p* < 0.05.

Abbreviations: LDS, E.max CAD; PICN, VITA Enamic; RNC, Lava Ultimate; YTZP, inCoris ZI.

**Figure 3:**
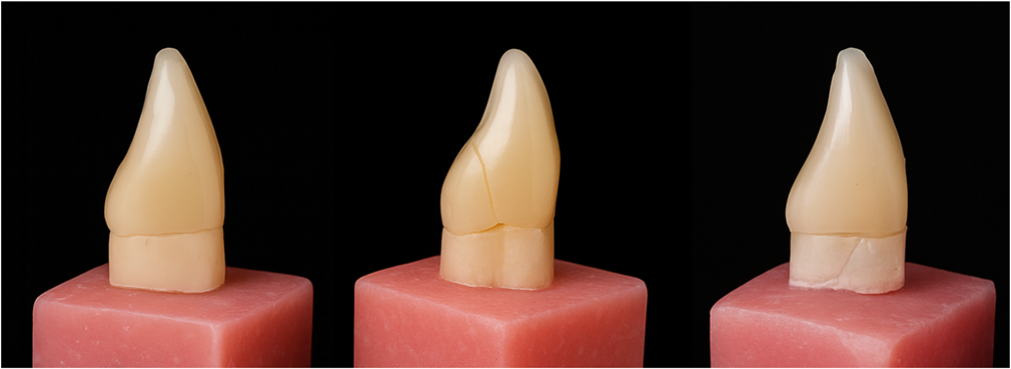
Representative examples of failure modes in restored teeth. From left to right: post‐crown decementation, repairable coronal fracture, and non‐repairable vertical root fracture.

Repairable (post/coronal fractures above the cemento–enamel junction) and non‐repairable (vertical root or catastrophic) failures were observed in all post‐crown groups (Table 3), with the PICN group showing the fewest non‐repairable failures (Figure 3).

**Table 3:** Types of failures within the groups based on their repairability.

Group	Repairable	Nonrepairable
Control	8	2	
LDS	8	2	
YTZP	5	5	
RNC	8	2	
PICN	9	1	

Abbreviations: LDS, E.max CAD; PICN, VITA Enamic; RNC, Lava Ultimate; YTZP, inCoris ZI.

## 4 Discussion

This study evaluated the fracture resistance of teeth with intact coronal structures versus those restored with single‐piece post‐crowns from four CAD–CAM materials, including failure nodes. Teeth with only a direct composite restoration after root canal access exhibited the highest fracture resistance. The YTZP single‐piece post‐crown group showed similar resistance to intact teeth. LDS had intermediate performance, not significantly different from YTZP, RNC, or PICN. The control and YTZP groups demonstrated higher resistance than the RNC or PICN. The null hypothesis was partially rejected. Despite similar fracture resistance, the YTZP group had more non‐repairable fractures (five) than the control (two). PICN and RNC, with lower resistance, predominantly failed in a repairable manner. This suggests that higher fracture resistance does not necessarily imply better failure behavior, as materials with lower strength can be advantageous for retreatment or repair. Clinically, fractures above the cemento–enamel junction are favorable as they can often be managed with adhesive restorations like composite buildups or bonded ceramics, preserving the tooth. Fractures of the post without root involvement allow for retreatment with a new post‐and‐core. Although materials such as PICN and RNC exhibit lower strength, their propensity for repairable failure offers practical advantages by simplifying management and extending tooth longevity. Large standard deviations in the control group may reflect anatomical variability in root canal and dentinal conditions before extraction (Mörmann et al. 2013).

Metal post‐cores have been reported to have shortcomings, such as post‐retention loss, post‐corrosion, aesthetic problems, and vertical root fractures (Mortazavi et al. 2012). Previous finite element analysis of ceramic posts showed reduced stress distribution in dentin due to an increased modulus of elasticity, which reduced dentin stresses as the forces were distributed to the post–tooth interface (Shetty et al. 2013). In this study, the fracture strengths of non‐metallic post‐crown materials were evaluated and compared.

Pissis (1995) developed a monoblock technique that involved fabricating a glass‐ceramic material. However, the fracture strength of this material was quite low. To address these structural issues, zirconia ceramic was introduced as a material for prefabricated endodontic posts (Bateli et al. 2014; Meyenberg et al. 1995; Özcan and Şahin 2013). In 2007, Awad and Marghalani (2007) and Streacker and Geissberger (2007) described techniques for milling a single‐piece zirconia post and core. In this study, alternative aesthetic materials were utilized for single‐piece post‐crown fabrication, and the results may be more reliable due to significant improvements in the materials since the earlier studies. The fracture strengths of all groups using the proposed post‐crown materials exceeded 222 N, which Bakke et al. (1990). previously described as the average force relevant to post‐applications in anterior teeth. The findings from a study by Bittner et al. (2010) also support those of the current study, as the fracture resistance of zirconia single‐piece milled post‐cores surpassed the required average. Shahrbaf et al. (2014) noted that all milled ceramic crowns fabricated on maxillary premolars showed higher fracture strengths than the average occlusal force applied to maxillary premolars, which is 300 N. This study yielded similar results for single‐piece post‐crown fracture resistance, with all groups displaying higher mean fracture resistance values than the average functional forces.

A prior study examining the mechanical properties of zirconium posts reported high fracture and bending strengths, similar to this study. The previous research identified the YTZ‐P‐based post‐crown group as recording the highest fracture strengths after the control group (Christel et al. 1989). Consistent with these findings, YTZP also showed the highest fracture strength in this study, outperforming RNC and PICN significantly. While YTZP often failed catastrophically, RNC and PICN had a higher proportion of repairable failures, suggesting that the modulus of elasticity influences both load‐bearing capacity and failure mode.

Understanding the fracture behavior of dental materials can be better understood by looking at their elastic moduli. YTZP, with its high modulus of 200–210 GPa, resists deformation and mimics natural dentition in strength (de Andrade et al. 2019). LDS, with a moderate modulus of 95–105 GPa, offers intermediate fracture resistance (de Andrade et al. 2019). Materials like PICN (30–40 GPa) and RNC (10–15 GPa) have elastic properties closer to dentin, facilitating stress distribution but with lower load‐bearing capacity; they fracture at lower loads (de Andrade et al. 2019; Beji Vijayakumar et al. 2021; Hong et al. 2021). Interestingly, failures in PICN and RNC are more repairable, which is advantageous clinically. Therefore, both mechanical strength and elastic modulus are critical in evaluating fracture resistance and repair potential, with PICN and RNC offering benefits for tooth preservation despite lower strength (de Andrade et al. 2019; Beji Vijayakumar et al. 2021; Hong et al. 2021).

Vafaee et al. (2010) reported that the most likely initial fracture point occurs at the dentin/core material junction. The findings of our study regarding fracture types and failures were consistent with the results of that study. In a previous investigation of PICNs, researchers demonstrated mechanical properties similar to those of human dentin and enamel (Coldea et al. 2013). In the current study, nearly all PICN fracture failure types were repairable, possibly due to the similarity in elastic modulus between natural teeth and PICN material. Another study evaluating the mechanical properties of PICNs reported their fracture resistance as 1.09 ± 0.05 MPa, which sits between those of ceramics and resin‐based composites; however, the authors cautioned that this data should be interpreted carefully due to factors related to their test design (Della Bona et al. 2014). In the present research, the fracture strength of the PICN group was significantly lower than that of YTZP and numerically lower than LDS, though not statistically different from LDS. A study assessing the Martens hardness of various materials, including YTZ‐P‐based ceramic, lithium disilicate ceramic, PICN, and RNC, found that YTZ‐P‐based ceramic was the hardest, followed in descending order by lithium disilicate, PICN, and RNC (Mörmann et al. 2013). In this study, the fracture strengths of these materials also decreased in the same order, suggesting a possible correlation with hardness.

Custom‐fabricated post cores demonstrate superior fracture resistance compared to prefabricated posts, attributable to their enhanced fit within the post space (Sendhilnathan and Nayar 2008; Zhi‐Yue and Yu‐Xing 2003). Consequently, they are particularly indicated for teeth exhibiting extreme coronal destruction, surpassing the application of prefabricated posts in such cases (Martinez‐Insua et al. 1998; Pierrisnard et al. 2002). To more accurately replicate the challenging clinical conditions, the post‐crown samples were prepared with a flat cut and tested without a ferrule. This methodological approach facilitated a more precise evaluation of the material types, eliminating the influence of the ferrule on the test outcomes (Milot and Stein 1992; Libman and Nicholls 1995).

This study had some limitations. It was an in vitro investigation that could not fully replicate oral conditions. The test used in this study was a static load applied at one point in a static pattern without aging. The study only evaluated the maxillary central incisors; therefore, the results can only be attributed to this group of teeth. Further studies with artificial aging and cyclic loading should be conducted, and clinical follow‐up studies should be performed to clarify the short‐ and long‐term successes of the materials tested in this experimental study under natural oral conditions and functional forces in vivo.

Choosing the most fracture‐resistant materials may not always be ideal from a clinical perspective. Although all the materials showed higher fracture resistance values expected in a clinical setting, the ease of repairing failures should also be part of treatment planning considerations. In this context, PICN and RNC, despite being weaker, might be better options when preserving tooth structure and preventing catastrophic root fractures are priorities.

## 5 Conclusions

All tested CAD/CAM–fabricated single‐piece post‐crowns exhibited fracture resistance exceeding the average functional forces encountered in the anterior region. YTZP demonstrated the highest strength, comparable to the control and LDS groups, and significantly greater than RNC and PICN. Notably, PICN tended to fracture in ways that are more easily repairable, with the highest incidence of such favorable failure modes. Consequently, when selecting a material for post‐crowns, clinicians should consider not only its fracture resistance but also the ease of repairing failures. In some cases, choosing a material with slightly lower strength but superior repairability may better preserve the natural tooth structure and facilitate future interventions.

## Author Contributions

Conceptualization: Ali Erdem and Erhan Dilber. Methodology: Ali Erdem, Erhan Dilber, Ebru Nur Işık, and Ibrahim Ersoy. Validation: Tan Fırat Eyüboğlu and Mutlu Özcan. Formal analysis: Ibrahim Ersoy. Investigation: Ali Erdem, Ebru Nur Işık, and Ibrahim Ersoy. Data curation: Ali Erdem, Ebru Nur Işık, and Ibrahim Ersoy. Writing – original draft preparation: Mehmet Selim Bilgin and Erhan Dilber. Writing – review and editing: Erhan Dilber, Tan Fırat Eyüboğlu, and Mutlu Özcan.

## Conflicts of Interest

The authors declare no conflicts of interest.

## Data Availability

The data are available from authors on request.
